# A209 ESOPHAGEAL EPIDERMOID METAPLASIA: ADVOCATING FOR A PRIMARY ENDOSCOPIC SUBMUCOSAL DISSECTION APPROACH

**DOI:** 10.1093/jcag/gwad061.209

**Published:** 2024-02-14

**Authors:** R Gilhotra, A Zarrin, S X Jiang, W Xiong, N Shahidi

**Affiliations:** The University of British Columbia Faculty of Medicine, Vancouver, BC, Canada; The University of British Columbia Faculty of Medicine, Vancouver, BC, Canada; The University of British Columbia Faculty of Medicine, Vancouver, BC, Canada; Department of Medicine, The University of British Columbia, Vancouver, BC, Canada; The University of British Columbia Faculty of Medicine, Vancouver, BC, Canada

## Abstract

**Background:**

Esophageal epidermoid metaplasia (EM), or leukoplakia, is a rare finding on endoscopy; typically seen incidentally as a white plaque and histologically characterized by orthokeratosis. The link between EM and squamous neoplasia has been identified. There is no consensus on the management of EM.

**Aims:**

To present a case of circumferential esophageal EM managed with endoscopic submucosal dissection (ESD).

**Methods:**

Case report and review of the literature

**Results:**

A 56-year-old female with a known history of EM was referred for consideration of ESD due to dysplastic progression confirmed on histopathology. Previous attempts at eradication included argon plasma coagulation and staged band mucosectomy. Fully circumferential ESD was performed over a 100mm segment in the mid esophagus using the double tunnel technique. Histopathology revealed multifocal low-grade squamous intraepithelial neoplasia within epidermoid metaplasia with negative margins.

**Conclusions:**

Esophageal epidermoid metaplasia may increase the risk of squamous cell carcinoma. There are no consensus recommendations regarding its management with previous described strategies including surveillance and eradication with radiofrequency ablation and band mucosectomy. ESD for epidermoid metaplasia has not been described; however, given the overall efficacy, efficiency, safety and now widespread availability of this technique a primary ESD approach should be considered.

**Funding Agencies:**

None

